# Endocrine-disrupting chemicals in male reproductive health: exposure risks, reproductive toxicity and emerging perspectives

**DOI:** 10.3389/fendo.2026.1956000

**Published:** 2026-10-05

**Authors:** Dongyue Ma, Qingjiang Yang, Jun Guo, Wenguang Zhou, Chen Huang, Xiangping Sun

**Affiliations:** 1 Guo Jun Qihuang Scholar Workshop, Ningxia Traditional Chinese Medicine Hospital and Research Institute of Traditional Chinese Medicine, Yinchuan, Ningxia, China; 2 Xiyuan Hospital of Clinical Medical College, Beijing University of Chinese Medicine, Beijing, China; 3 Department of Andrology, Xiyuan Hospital of China Academy of Chinese Medical Sciences, Beijing, China

**Keywords:** endocrine-disrupting chemicals, environmental health, male reproductive health, mixture exposure, reproductive toxicity

## Abstract

Male reproductive health represents a growing global health challenge, and increasing evidence highlights the contribution of environmental exposures to declining male reproductive function. Endocrine-disrupting chemicals are widespread environmental contaminants that interfere with endocrine regulation and have been associated with impaired semen quality, hormonal imbalance and testicular dysfunction. Here, we summarize current advances in understanding EDC-induced male reproductive toxicity, focusing on major chemical classes, including bisphenols, phthalates, pesticides, per- and polyfluoroalkyl substances and heavy metals. We discuss the interconnected mechanisms underlying reproductive impairment, including disruption of the hypothalamic-pituitary-gonadal axis, altered steroidogenesis, oxidative stress, mitochondrial dysfunction, blood-testis barrier disruption, immune dysregulation and epigenetic reprogramming. Importantly, emerging evidence from developmental origins of health and disease perspectives indicates that exposure during critical developmental windows may establish persistent molecular alterations, influencing reproductive outcomes across the life course. In addition, the metabolic effects of certain EDCs, including obesity and metabolic dysfunction, may further exacerbate male reproductive impairment. Given that human exposure occurs predominantly in the form of complex chemical mixtures, future research should integrate exposomics, multi-omics approaches and longitudinal cohorts to clarify causal relationships and identify susceptible populations. Such advances will be essential for developing precision environmental health strategies to preserve male reproductive function.

## 1 Introduction

cThe decline in male reproductive health has emerged as a major global public health concern, with growing evidence of declining semen quality and impaired reproductive function worldwide. Although male factors account for approximately 30–50% of infertility cases according to the World Health Organization, the etiology of male infertility remains incompletely characterized (1). Over recent decades, reported declines in sperm concentration and other semen parameters have heightened concerns about the environmental determinants of male reproductive health. Although genetic susceptibility, aging, lifestyle factors and medical conditions contribute to infertility risk, accumulating evidence indicates that widespread exposure to endocrine-disrupting chemicals (EDCs) is an important and potentially preventable environmental risk factor (2).

EDCs are a diverse group of exogenous chemicals that interfere with endocrine signaling by mimicking, antagonizing, or otherwise modifying endogenous hormonal activity. These compounds are ubiquitous in modern environments and are detected in food packaging, personal care products, medical devices, industrial materials and environmental media. Human exposure occurs continuously through multiple routes, including ingestion, inhalation and dermal absorption (3). Through interference with hormone-dependent signaling, EDCs may impair male reproductive function by altering hypothalamic–pituitary–gonadal (HPG) axis activity, steroidogenesis, testicular development and spermatogenesis (4).

EDC-induced male reproductive impairment involves a complex network of molecular events rather than a single pathological pathway. Beyond endocrine disruption, EDCs can promote oxidative stress, mitochondrial dysfunction, inflammatory responses, blood–testis barrier (BTB) disruption and epigenetic reprogramming, ultimately affecting sperm production and function (5, 6). Importantly, emerging evidence from developmental origins of health and disease perspectives suggests that exposure during sensitive developmental windows, including fetal life, childhood and adolescence, may establish persistent biological alterations that influence reproductive capacity across the life course. Moreover, epigenetic mechanisms may provide a basis for intergenerational or transgenerational effects, although evidence for true transgenerational inheritance in humans remains limited and requires further investigation (7).

In this review, we summarize current knowledge regarding the major classes of EDCs associated with male reproductive toxicity and their underlying molecular mechanisms. We further discuss the long-term consequences of EDC exposure during critical developmental windows, the challenges posed by real-world mixture exposures, and emerging approaches integrating exposomics and multi-omics technologies. By providing a mechanistic framework linking environmental exposure to reproductive dysfunction, this review aims to advance understanding of EDC-related male infertility and highlight future directions for prevention and precision environmental health strategies.

## 2 Methods

### 2.1 Search strategy

A systematic literature search was performed in Web of Science Core Collection from inception to June 2026. The search combined MeSH terms and free-text keywords with Boolean operators, using the following core string: (“endocrine disrupt*” OR “endocrine disruptors” OR “endocrine disruptor chemicals” OR “EDC” OR “endocrine disrupting chemicals”) AND (“sperm*” OR “spermatozoa” OR “spermatozoon” OR “male reproductive health” OR “male fertility” OR “testicular” OR “spermatogenesis”). Additional targeted searches were run for specific EDC classes (bisphenols, phthalates, PFAS, pesticides, heavy metals) and individual reproductive endpoints. Reference lists of relevant reviews and authoritative reports were hand-searched for supplementary studies.

### 2.2 Inclusion and exclusion criteria

Eligible studies included: (1) original research (experimental, epidemiological, or biomonitoring); (2) investigating exposure to at least one EDC; (3) reporting outcomes related to male reproductive health (semen quality, hormone levels, testicular function, or congenital anomalies).

Exclusion criteria were: systematic reviews, meta-analyses, and narrative reviews, conference abstracts, editorials, case reports, duplicate publications, and studies focusing solely on female reproductive outcomes without male-relevant data.

### 2.3 Study selection and data extraction

The initial database search yielded 1,633 records. After limiting to the “article” document type, 1,163 records remained for screening. Two reviewers (Yang Qingjiang and Ma Dongyue) independently screened the titles and abstracts against the predefined eligibility criteria. Full-text assessment of the remaining articles was subsequently performed by the same two reviewers, with discrepancies resolved through discussion or consultation with a third reviewer (Zhou Wenguang), who also oversaw data verification. A final set of 56 studies met all inclusion criteria and were included in the present review (**Table 1**). From each eligible study, the following data were extracted: study design, population and sample characteristics, types of EDCs measured, reproductive outcomes assessed, and main findings.

**Table 1:** Common EDCs and their reproductive toxic effects.

Evidence type	Subject	Compound	Dose	Main mechanism	Male reproductive toxicity	Ref.
Human epidemiological study	The urine and semen of 218 male individuals	BPA	NA	NA	Elevated urinary BPA showed dose-dependent negative associations with reduced sperm concentration, total sperm count and sperm vitality	(50)
Human epidemiological study	The urine and semen of 190 infertile-clinic men	BPA	NA	NA	Suggestive declines in sperm concentration, motility, morphology; increased sperm DNA fragmentation	(51)
Human prospective epidemiological study	375 fertile men	BPA	NA	NA	Urinary BPA positively associated with SHBG, inversely associated with free androgen index (FAI) and FAI/LH ratio	(52)
Human occupational cohort study	153 male offspring	BPA	NA	NA	Shortened anogenital distance (AGD) in boys	(53)
In-vivo animal study	ICR male mice	BPA	5-200 mg/kg bw, intragastric administration, 45 d	Induces spermatogenic ferroptosis through oxidative stress, iron overload, and mitochondrial damage	Reduced testicular weight, decreased sperm concentration and motility, increased sperm malformation rate	(54)
In-vivo animal study	ICR male mice	BPA	50 mg/kg bw/day, oral gavage, 6 weeks	Disrupts histone to protamine transition, alters histone variants and modifications, elevates sperm DNA methylation, interferes with estrogen signaling	Decreased sperm concentration and motility, reduced hyperactivation, decreased fertilization and blastocyst formation rates	(55)
Experimental animal study	9-week-old Wistar rats	BPA	0, 2, 20, 200 μg/kg/day by gavage for 60 days	ER-mediated meiotic disruption, persistent meiotic DNA double-strand breaks in pachytene spermatocytes, activation of ATM/Chk2 signaling, and germ-cell apoptosis	Impaired spermatogenesis manifested as reduced epididymal sperm count, disrupted meiosis I, and increased apoptosis	(17)
Experimental animal study	Pregnant ICR mice and male offspring	BPA	50 mg/kg/day, ED0.5–18.5	Impaired Leydig-cell steroidogenesis, with downregulation of StAR, CYP11A1 and CYP17A1, together with increased Leydig-cell apoptosis	Decreased serum testosterone, reduced testicular weight, impaired spermatogenesis, decreased sperm count and motility, and reduced male fertility	(18)
Human epidemiological study	796 healthy male college students	13 urinary phthalate metabolites	NA	NA	Phthalate exposure was associated with impaired semen quality and altered reproductive hormones	(56)
Human cross-sectional epidemiological study	379 men from infertility clinic	Phthalate metabolites	NA	NA	MEP, MBP, MBzP associated with elevated sperm DNA damage	(57)
Human cross-sectional and in-vitro experimental study	180 fertile men and purified human spermatozoa	DEHP, DBP	NA	NA	Seminal DEHP/DBP negatively correlated with sperm motility among infertile men in-vitro and In-vivo	(58)
Human epidemiological study	Urine and semen samples from 463 adult men attending an infertility clinic	Phthalate metabolites	NA	NA	Higher urinary MBP and MBzP concentrations were associated with reduced sperm concentration, total motile sperm count, sperm motility, and abnormal sperm morphology	(59)
Human epidemiological study	425 adult men from an infertility evaluation cohort	DEHP metabolites	NA	NA	Higher urinary DEHP metabolites were significantly associated with lower serum total testosterone, estradiol, and FAI	(60)
Human epidemiological study	6,246 newborn boys, including 102 cryptorchidism cases	Phthalate	NA	NA	Maternal self-reported phthalate exposure was positively associated with cryptorchidism	(61)
In-vivo animal study	Wistar rats	DEHP	Prenatal GD13-20 oral 750 mg/kg bw/day; post-natal puberty PND23-53 oral 750 mg/kg bw/day	Anti-androgen effect	Prenatal DEHP exposure significantly shortens male rat AGI	(62)
Experimental animal study	Pregnant Wistar rats and male offspring	Phthalate	500 mg/kg/day, GD13-21	Impairs spermatogenesis, steroidogenesis, decreased testis size	>60% cryptorchidism, hypospadias, infertility and testicular abnormalities in male offspring	(63)
Experimental animal study	Pregnant Wistar rats and male offspring	DBP	300 or 900 mg/kg/day	Delays testicular development and decreased testosterone secretion during the critical urethral-development window	Hypospadias incidence 22.7% at 300 mg/kg and 43.5% at 900 mg/kg; cryptorchidism 17.4% at 900 mg/kg	(64)
Experimental animal study	Pregnant Sprague-Dawley rats and F1 male offspring	DBP	250, 500, 700 mg/kg/day, GD10-19	Reduces androgen signaling, decreases testosterone and dihydrotestosterone, downregulates androgen receptor and 5-α-reductase type 2	Dose dependent increases in hypospadias and cryptorchidism, reduced testicular and accessory sex organ weights, decreased anogenital distance	(65)
Experimental animal study	Pregnant rats and male offspring	MBP	Maternal gavage, GD15-18	Sertoli-cell dysfunction and disturbed Müllerian-inhibiting substance signaling	84.6% cryptorchidism in male offspring versus 0% in controls; abnormal transabdominal testicular ascent	(66)
Experimental animal study	Pregnant rats and male offspring	DBP	250–1000 mg/kg/day, GD14-18	Suppression of androgen-dependent reproductive development and testosterone production	Hypospadias incidences were 6.8% (500 mg/kg) and 41.3% (750 mg/kg), with concurrent cryptorchidism and reduced AGD	(67)
Experimental animal study	Pregnant rats and male offspring	DBP	850 mg/kg/day, GD11-15	Reduced testosterone and down-regulation of Cyp11a1, Hsd3b, Scarb1, Star, AR, Srd5a2; disruption of Shh/Fgf/Wnt developmental signaling	Hypospadias, testicular dysplasia and combined urogenital malformations	(68)
Human cross-sectional occupational study	31 organophosphate pesticide sprayers, 80 unexposed control men	Organophosphate pesticides (OPs)	NA	NA	Lower semen quality parameters including volume, motility, normal morphology, and hormone levels; elevated liquefaction time, pH, immature sperm, and leukocytes	(69)
Human nested case-control study	94 cases (low semen quality) and 95 controls (normal semen quality)	OPs	NA	NA	Higher urinary dimethyl-phosphate (DMP) was associated with elevated odds for low sperm concentration/motility	(70)
Human epidemiological study	356 men aged 20–55 years from the U.S. general population	OPs	NA	NA	Urinary diethyl phosphate (DEP) was significantly inversely associated with serum testosterone, suggesting that exposure to certain OP insecticides may alter androgen homeostasis in adult men	(71)
Human occupational epidemiological study	Male workers occupationally exposed to organophosphate pesticides	Parathion, methyl parathion and methamidophos	NA	NA	OP pesticide exposure was associated with increased serum LH and decreased testosterone, with a smaller increase in FSH	(72)
Experimental animal study	Male Wistar rat model	Malathion	0, 33.75, 54, and 108 mg/kg, oral gavage for 60 days	Induces testicular oxidative and endocrine toxicity, increases spermatogenic cell apoptosis, alters testicular enzymes, impairs reproductive hormone production	Decreased sperm count and motility, seminiferous tubule degeneration, reduced serum LH, FSH and testosterone levels	(73)
Experimental animal study	Adult male Wistar rats	Chlorpyrifos	2.7, 5.4 and 12.8 mg/kg/day, oral gavage for 90 days	Disrupts testicular function and steroid hormone homeostasis, impairs spermatogenesis	Reduced testicular sperm count and motility, increased sperm abnormalities, seminiferous tubule degeneration, decreased testosterone levels, elevated FSH levels	(74)
Experimental animal study	Male Wistar rats, postnatal day 25	Malathion	10 or 50 mg/kg/day, oral gavage for 40 days	Downregulation of testicular AR and 17β-HSD, disturbance of androgen signaling and antioxidant defense	Reduced sperm count, increased sperm abnormalities, impaired spermatogenesis, compromised acrosomal integrity; high dose also decreased plasma testosterone	(75)
In-vivo animal study	Adult male Wistar rats	chlorpyrifos	3.0 mg/kg bw/day, gavage, 20 weeks	Inhibits acetylcholinesterase, disrupts hypothalamic pituitary gonadal axis, induces testicular oxidative stress	Decreased serum LH, FSH and testosterone, testicular oxidative damage, reduced sperm count, increased sperm abnormality rate, seminiferous tubule injury	(76)
Human epidemiological study	247 healthy young men, median age 19 years	PFOS and 13 other PFCs	NA	NA	Serum PFOS was significantly negatively associated with total testosterone, calculated free testosterone, free androgen index, and ratios of these hormones to LH	(77)
Human epidemiological study	651 adult men from Nanjing, China	PFOA, PFNA, PFOS and 6:2 Cl-PFESA	NA	NA	PFAS exposure correlated with reduced total and free testosterone, lower SHBG, and decreased testosterone to LH ratio	(78)
Human epidemiological study	902 adult men, mean age 31.3 years	24 PFAS including PFOS and PFOA isomers and emerging PFAS such as F-53B components	NA	NA	PFAS mixture exposure was associated with altered total testosterone, estradiol, FSH, LH, SHBG and INSL3	(79)
Human occupational epidemiological study	111 workers in 1993 and 80 workers in 1995	PFOA	NA	NA	No significant association between serum PFOA and testosterone; modest estradiol increase in highly exposed workers, but confounded by BMI	(80)
Human cross-sectional epidemiological study	103 male participants	PFAAs	NA	NA	Seminal PFAAs and individual PFAAs (PFOA, PFOS) negatively correlated with progressive sperm motility	(81)
Human prospective birth-cohort study	500 male infants	PFASs	NA	NA	Reduced AGD at birth and 6 months	(82)
Experimental animal study	8-week-old male CD-1 mice	PFOS	1, 5 or 10 mg/kg/day, oral gavage for 7, 14 or 21 days	Disrupts testicular endocrine signaling, downregulates StAR, CYP11A1, CYP17A1, 3-β-HSD and 17-β-HSD	PFOS significantly decreased serum testosterone and epididymal sperm count, with marked reduction in steroidogenic enzyme expression	(83)
Experimental animal study	Pregnant Sprague-Dawley rats and fetal testes	PFOS	5 or 20 mg/kg, oral gavage, gestational days 11 to 19	Impairs fetal Leydig cell development and steroidogenesis, reduces cholesterol availability and steroidogenic capacity	Reduced fetal Leydig cell number and function, decreased testosterone production	(84)
Experimental animal study	Adult ICR mice and primary mouse Leydig cells	PFOS	0 to 10 mg/kg/day, gavage for 4 weeks; in vitro Leydig cell exposure	Disrupts p38, PKA, CREB, CRTC2 and StAR signaling; suppresses CREB and CRTC2 binding to StAR promoter	Dose dependent decrease in testosterone and sperm count, damaged testicular interstitium, suppressed testosterone biosynthesis	(85)
Experimental animal study	Male BALB/c mice, 6 to 8 weeks old	PFOA	0.31, 1.25, 5 or 20 mg/kg/day, gavage for 28 days	Directly disturbs testicular steroidogenic machinery, impairs Leydig cell function and CYP11A1 and 17-β-HSD signaling	Dose dependent reductions in testicular testosterone and progesterone, seminiferous tubule injury, impaired sperm quality, decreased CYP11A1 and INSL3	(86)
Experimental animal/*in vitro* study	Primary immature rat Leydig cells and 35-day-old rats	PFOA	In vitro at 0.01, 0.1 and 1 micromolar; in vivo at 5 or 10 mg/kg	Inhibits androgen biosynthesis via downregulation of LHCGR, CYP11A1, 3-β-HSD1 and NR5A1, and inhibits CYP11A1 and 3-β-HSD1 activities	Reduced total androgen biosynthesis in immature Leydig cells, decreased serum androgen levels in rats	(87)
Experimental animal study	Fetal rat testes and adult rat seminiferous tubules	PFOA	0 to 100 micrograms per milliliter for 24 hours	Suppresses StAR dependent steroidogenesis and induces testicular cell injury	Reduced cAMP, progesterone, testosterone and StAR protein in fetal testis cultures	(88)
Experimental animal study	Adult male rats	PFDoA	1, 5 or 10 mg/kg/day, oral exposure for 14 days	Suppresses cholesterol transport and steroidogenic gene expression, induces Leydig, Sertoli and germ cell apoptosis	Significantly decreased testosterone at 5 and 10 mg/kg/day, altered expression of multiple steroidogenesis related genes, caused testicular ultrastructural injury and apoptosis	(89)
Human epidemiological study	Chinese male college students	Pb	NA	NA	Urinary Pb negatively associated with testosterone, FSH and testosterone to LH ratio; reduced sperm concentration, total count, progressive and total motility	(90)
Human epidemiological study	Adult men from general population	Cd	NA	NA	Urinary Cd showed dose response with elevated testosterone, but not an independent determinant of testosterone, FSH or LH	(91)
Human epidemiological study	60 infertile and 40 fertile men	Cd	NA	NA	Higher Cd linked to azoospermia or oligozoospermia, poorer semen quality and altered FSH	(92)
Human epidemiological study	Chinese men aged 18 years and above	Cd	NA	NA	Each doubling of blood Cd linked to 5.04 percent increase in testosterone and 4.03 percent increase in testosterone to estradiol ratio	(93)
Human epidemiological study	61 men attending infertility clinics	Pb, Cd and Hg	NA	NA	Increased sperm immotility associated with seminal Pb and Cd	(94)
Human epidemiological study	529 male partners of pregnant women	Hg	NA	NA	Blood Hg was positively associated with inhibin B, particularly in Greenlandic men, but associations with testosterone, FSH, and LH were inconsistent, providing limited evidence for Hg-induced androgen suppression	(95)
Human occupational study	240 men without known occupational metal exposure	Pb	NA	NA	Low level Pb linked to increased abnormal and immature sperm and altered hormones including elevated testosterone and estradiol; Pb and Cd showed synergistic effects on testosterone	(96)
Experimental animal study	Adult male rats	Pb	0.1 or 0.3 percent lead acetate in drinking water for 30 days	Suppresses testicular steroidogenesis, disrupts hypothalamic pituitary testicular axis	Blood Pb negatively correlated with serum and intratesticular testosterone; decreased intratesticular sperm count; suppressed serum FSH	(97)
Experimental animal study	Adult male rats	Pb	8 mg/kg/day, i.p., 35 days	Leydig cell dysfunction and impaired steroidogenesis	Plasma and testicular testosterone decreased by approximately 80%, and the testosterone-to-LH ratio was markedly reduced	(98)
Experimental animal study	Male BALB/c mice	Pb	Lead acetate in drinking water (366 mg/L, ~0.97 mg Pb/animal/day) for 6 months	Inhibits intratesticular androgen biosynthesis	Intratesticular testosterone decreased significantly after 30 days, while androstenedione was reduced at later time points	(99)
Experimental cell study	Primary rat Leydig cells	Cd	10, 20 and 40 micromolar cadmium chloride for 24 hours	Direct Leydig cell cytotoxicity, oxidative stress, DNA damage, impaired steroidogenesis	Leydig cell viability and testosterone secretion were significantly reduced, accompanied by increased MDA and DNA damage, as well as altered SOD and GSH-Px activities	(100)
Experimental cell study	Leydig cells from KP and CBA mice	Cd	Single CdCl_2_ injection	Inhibits androgen biosynthesis and 17-β-HSD activity	Marked decrease in androgen production, reduced 17-β-hydroxysteroid dehydrogenase and other oxidoreductive enzyme activities	(101)
Experimental animal study	Pregnant rats and male offspring	Pb and Cd	0.05 mg/kg/day lead acetate, cadmium acetate or combined exposure throughout gestation and lactation	Inhibits testicular steroidogenic enzymes, induces oxidative stress, depletes cholesterol and GSH	Significantly reduced steroidogenic enzyme activities and serum testosterone, altered sperm count and motility, decreased SOD, CAT and GSH, increased lipid peroxidation	(102)
Experimental animal study	Adult male Wistar rats	Pb, Cd and Hg	Low dose mixture of lead chloride, cadmium chloride and mercury chloride	Induces oxidative stress and lipid peroxidation, disrupts HPT axis and testicular endocrine function	Decreased testosterone, LH and FSH, reduced epididymal sperm count and viability, increased abnormal sperm morphology, decreased SOD, CAT and GSH, increased testicular MDA	(103)

6:2 Cl-PFESA, 6:2 chlorinated polyfluorinated ether sulfonate; AGD, anogenital distance; AGI, anogenital index; AR, androgen receptor; BPA, bisphenol A; cAMP, cyclic adenosine monophosphate; CAT, catalase; CPF, chlorpyrifos; CREB, cAMP response element-binding protein; CRTC2, CREB-regulated transcription coactivator 2; CYP11A1, cytochrome P450 11A1 (cholesterol side-chain cleavage enzyme); CYP17A1, cytochrome P450 17A1; DBP, di-n-butyl phthalate; DEHP, di(2-ethylhexyl) phthalate; DEP, diethyl phosphate; DMP, dimethyl phosphate; F-53B, a commercial chlorinated polyfluorinated ether sulfonate mixture; FAI, free androgen index; FSH, follicle-stimulating hormone; GD, gestational day; GSH, glutathione; GSH-Px, glutathione peroxidase; HPG axis, hypothalamic-pituitary-gonadal axis; HPT axis, hypothalamic-pituitary-testicular axis; HSD, hydroxysteroid dehydrogenase (e.g., 3β-HSD, 17β-HSD); INSL3, insulin-like peptide 3; LH, luteinizing hormone; LHCGR, luteinizing hormone/choriogonadotropin receptor; MBP, monobutyl phthalate; MBzP, monobenzyl phthalate; MDA, malondialdehyde; MECPP, mono(2-ethyl-5-carboxypentyl) phthalate; MEHHP, mono(2-ethyl-5-hydroxyhexyl) phthalate; MEHP, mono(2-ethylhexyl) phthalate; MEOHP, mono(2-ethyl-5-oxohexyl) phthalate; MEP, monoethyl phthalate; NR5A1, nuclear receptor subfamily 5 group A member 1; OPs, organophosphate pesticides; Pb, lead; PFAS, per- and polyfluoroalkyl substances; PFAAs, perfluoroalkyl acids; PFDoA, perfluorododecanoic acid; PFNA, perfluorononanoic acid; PFOA, perfluorooctanoic acid; PFOS, perfluorooctane sulfonate; PKA, protein kinase A; PND, postnatal day; SHBG, sex hormone-binding globulin; SOD, superoxide dismutase; StAR, steroidogenic acute regulatory protein.

## 2 Major categories of EDCs and their reproductive toxicity

EDCs are exogenous compounds capable of interfering with the normal function of the endocrine system. They are widespread in the environment, food, industrial products, and consumer goods. With escalating environmental contamination and increased human exposure, the potential threats of EDCs to male reproductive health are of growing concern. Epidemiological and experimental studies consistently demonstrate that EDCs impair male fertility through multiple pathways, including endocrine disruption and direct reproductive toxicity, thereby disrupting the testicular microenvironment and impairing spermatogenesis (**Figure 1**). First, EDCs can bind to estrogen receptors (ERs), androgen receptors (ARs), or aryl hydrocarbon receptors (AhRs), mimicking or antagonizing endogenous hormones and disrupting steroidogenesis and signaling pathways, thereby impairing germ cell proliferation and differentiation in the testes (8). Second, many EDCs induce mitochondrial dysfunction, leading to energy metabolism disorders, apoptosis, and disruption of spermatogenesis (9). Third, some EDCs compromise the BTB and alter the function of Sertoli and Leydig cells, resulting in local inflammation, immune imbalance, and sperm DNA damage (10, 11). Moreover, increasing evidence suggests that adverse EDC effects may involve epigenetic mechanisms, which not only deteriorate sperm quality in exposed individuals but also exert transgenerational effects on offspring reproductive health (12).

**Figure 1:**
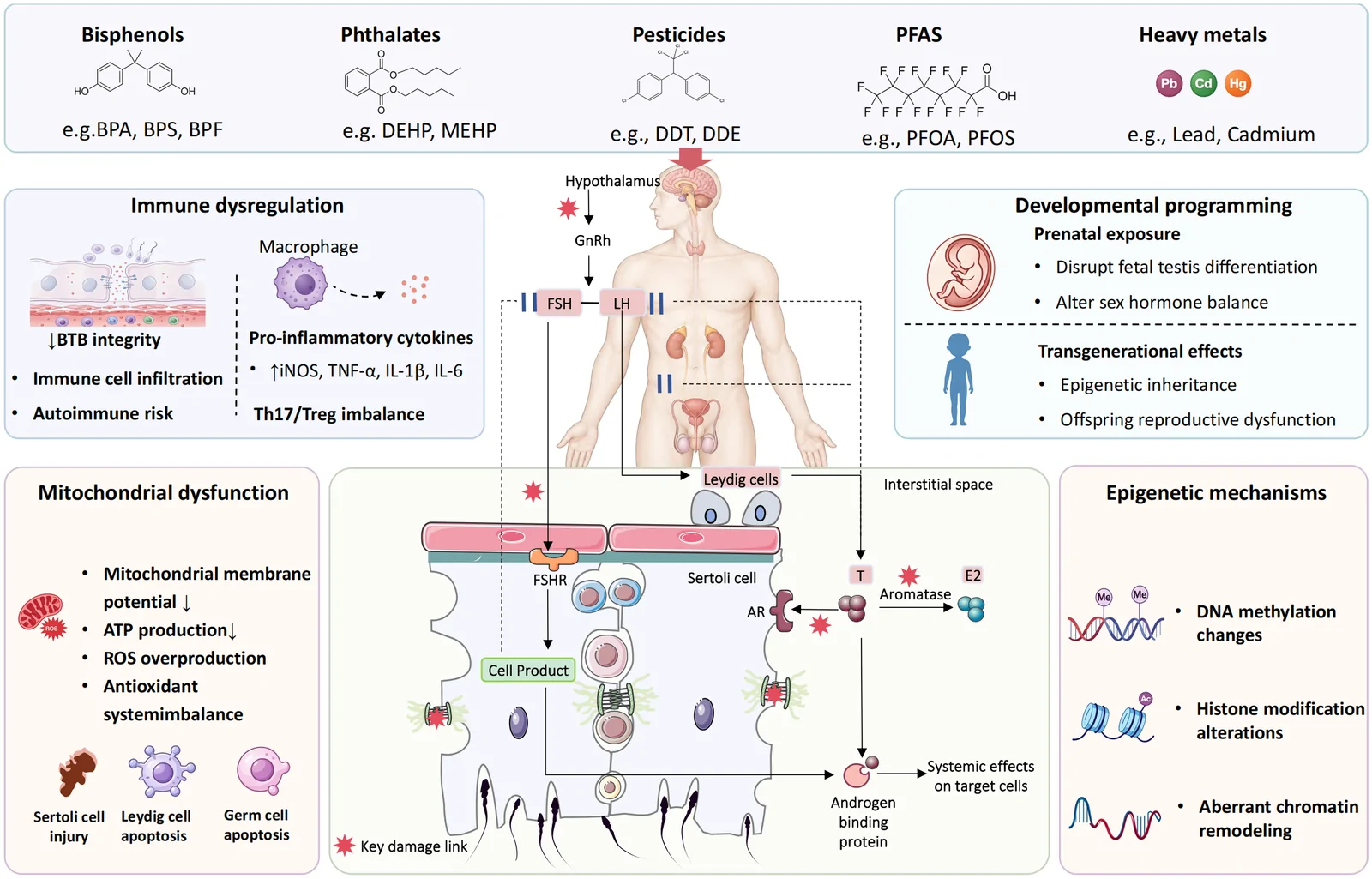
Major pathways of EDC-induced male reproductive toxicity. BPA, bisphenol A; BPS, bisphenol S; BPF, bisphenol F; DEHP, di(2-ethylhexyl) phthalate; MEHP, mono(2-ethylhexyl) phthalate; DDT, dichlorodiphenyltrichloroethane; DDE, dichlorodiphenyldichloroethylene; PFAS, per- and polyfluoroalkyl substances; PFOA, perfluorooctanoic acid; PFOS, perfluorooctane sulfonate; BTB, blood–testis barrier; iNOS, inducible nitric oxide synthase; TNF-α, tumor necrosis factor-alpha; IL-1β, interleukin-1 beta; IL-6, interleukin-6; Th17, T helper 17 cell; Treg, regulatory T cell; ATP, adenosine triphosphate; ROS, reactive oxygen species; E2, estradiol; FSHR, follicle-stimulating hormone receptor; AR, androgen receptor.

Among bisphenols, bisphenol A (BPA) is one of the most common EDCs, widely used in the production of plastics and resins. Extensive epidemiological and experimental studies have shown that BPA exposure is closely associated with male infertility, reduced semen quality, and abnormal hormone levels. Zhang et al. reported that BPA induces OS and apoptosis in testicular interstitial cells of mice (13). Ryū et al. demonstrated in mice that BPA disrupts reproductive hormone levels and impairs testicular mitochondrial function, particularly energy production and apoptosis-related pathways, thereby contributing to spermatogenic failure and reduced fertilization capacity (14). Recent studies have expanded these findings by showing that BPA exposure can alter the testicular proteome and proteins involved in spermatogenesis, sperm structure, membrane integrity, and oxidative-stress response (15). In addition, BPA-induced mitochondrial dysfunction and lipid peroxidation may promote ferroptosis, with disturbances in GPX4, ACSL4, FTH1 and other ferroptosis-related pathways contributing to testicular injury and impaired sperm quality (16). BPA exposure has also been shown to disrupt meiotic progression during spermatogenesis through estrogen-like activity. In adult male rats, BPA interfered with meiotic progression, induced persistent meiotic DNA double-strand breaks, and increased germ-cell apoptosis, resulting in impaired spermiation and reduced sperm count, even in the absence of significant changes in circulating testosterone, LH, or FSH levels. These findings suggest that BPA may exert direct testicular toxicity through estrogen receptor-mediated disruption of meiosis (17). Moreover, prenatal BPA exposure can impair Leydig-cell development and steroidogenesis in male offspring, with downregulation of key steroidogenic factors, including StAR, CYP11A1, CYP17A1, HSD3B1, and HSD17B3. This impairment is accompanied by decreased serum testosterone, reduced testicular weight, decreased sperm count and motility, impaired spermatogenesis, and reduced male fertility (18). With growing recognition of BPA’s hazards, many manufacturers have introduced substitutes such as bisphenol S (BPS) and bisphenol F (BPF). However, these analogs retain endocrine-disrupting activity and interfere with spermatogenesis through mechanisms similar to BPA, raising increasing concern about their reproductive toxicity (19, 20).

Phthalates are widely used synthetic chemicals, primarily as plasticizers, and are found in plastics, cosmetics, food packaging, and a broad range of consumer products. Increasing epidemiological and experimental evidence suggests that phthalate exposure is strongly associated with impaired male reproductive function. Epidemiological studies have reported significantly elevated levels of phthalate metabolites in the serum and urine of exposed men, which are negatively correlated with testosterone synthesis, sperm count, motility, and morphology (21, 22). Mechanistically, mono-2-ethylhexyl phthalate (MEHP), an bioactive metabolite of di(2-ethylhexyl) phthalate, induces mitochondrial dysfunction and oxidative stress, leading to excessive ROS production and disruption of spermatogenesis (23). Phthalates may also impair cell junctions and compromise BTB integrity, aggravating germ cell damage (24). However, not all epidemiological studies have identified significant associations between phthalate exposure and semen quality, highlighting the potential influence of genetic susceptibility, exposure dose and duration, and lifestyle factors such as diet and smoking (25).

Pesticides, herbicides, and insecticides are pervasive environmental contaminants, primarily including organochlorines (OCs), organophosphates (OPs), and pyrethroids. Human exposure occurs through multiple routes, with occupational exposure particularly relevant in agricultural settings and non-occupational exposure arising from domestic applications and contaminated food or environmental media. These compounds have been associated with impaired semen quality and altered male reproductive function. Organochlorine pesticides—including dichlorodiphenyltrichloroethane (DDT), dichlorodiphenyldichloroethylene (DDE), methoxychlor, chlordane, hexachlorobenzene, and lindane—are among the most extensively studied. DDT exhibits estrogenic activity, while its metabolite DDE acts as an androgen receptor antagonist (26). Elevated serum levels of these compounds have been associated with reduced testosterone concentrations, lower sperm count and motility, and increased sperm DNA damage (27, 28). Although DDT and several OCs have been banned or restricted in many countries, their continued use in some malaria-endemic regions, together with their environmental persistence, remains a concern (29). Organophosphate pesticides, including chlorpyrifos, malathion, and dichlorvos, remain important agricultural contaminants, although the use of several compounds has been restricted because of their neurotoxicity (30). Elevated urinary levels of OP metabolites are commonly detected among occupationally exposed individuals and have been significantly associated with reduced sperm count and motility, abnormal morphology, and increased DNA fragmentation (31, 32). The underlying mechanisms mainly involve oxidative stress, reduced antioxidant enzyme activity, dysregulation of gonadal hormone secretion, and thyroid dysfunction (33). Pyrethroids, including cypermethrin, deltamethrin, and lambda-cyhalothrin, are widely used insecticides whose increasing application has raised concerns regarding potential reproductive toxicity (34). Although current evidence remains limited, studies have shown that higher urinary concentrations of the pyrethroid metabolite 3-phenoxybenzoic acid (3-PBA) are associated with decreased sperm concentration, increased DNA fragmentation, and abnormal sperm chromatin structure (35, 36). Collectively, pesticides may impair male reproductive function through convergent endocrine, oxidative, and genotoxic mechanisms, including perturbation of sex hormone signaling, steroidogenesis, and sperm genomic integrity (**Table 1**).

Per- and polyfluoroalkyl substances (PFAS) are synthetic organic compounds widely used in industry and consumer products, including water-repellent coatings, non-stick cookware, firefighting foams, food packaging, and textiles. Representative compounds include perfluorooctanoic acid (PFOA), perfluorooctane sulfonate (PFOS), and their derivatives (37). Owing to their highly stable carbon–fluorine bonds, PFAS are highly persistent in the environment and can bioaccumulate in biological systems, raising substantial concerns regarding their endocrine and reproductive toxicity (38, 39). Evidence from experimental and epidemiological studies suggests that PFAS exposure can adversely affect male reproductive function through multiple biological pathways. PFAS may impair Leydig cell function by altering the expression or activity of steroidogenic enzymes, thereby reducing androgen production and potentially disrupting spermatogenesis (38). This has been demonstrated in both animal studies and epidemiological cohorts, where occupational or environmental PFAS exposure was associated with decreased testosterone levels and semen quality (40). Second, PFAS may remodel the testicular immune microenvironment, promoting inflammatory responses and immune imbalance that further damage spermatogenesis (41). Notably, PFAS toxicity extends beyond adult males, raising concerns about sensitive developmental stages. Childhood and adolescence exposures have been linked to delayed puberty, genital abnormalities, and disordered sexual maturation, thereby increasing later-life infertility risk (42). Moreover, prenatal PFAS exposure alters maternal hormone homeostasis and disrupts fetal sexual differentiation and reproductive organ development, posing intergenerational risks to male reproductive health (43).

Certain heavy metals represent an important group of environmental reproductive toxicants, some of which may exert endocrine-disrupting activity. Among them, lead (Pb), cadmium (Cd) and mercury (Hg) are most frequently investigated in male reproductive toxicology. Importantly, heavy metals should not be regarded as a homogeneous group of endocrine-disrupting chemicals, as their reproductive toxicity may arise from both direct non-endocrine effects and endocrine-mediated mechanisms. Chronic exposure to Pb and Cd significantly increases the risk of infertility, being strongly associated with reduced sperm count, impaired motility, abnormal morphology, and DNA fragmentation (44). Beyond direct testicular toxicity mediated by oxidative stress, mitochondrial dysfunction, and genotoxicity, some heavy metals can also disrupt male reproductive endocrinology through multiple levels of the HPG axis (45). At the central level, Pb and other metals may interfere with hypothalamic and pituitary function, altering the secretion of gonadotropin-releasing hormone and gonadotropins (46). At the testicular level, metals can directly impair Leydig-cell function and steroidogenesis by inducing oxidative stress and interfering with steroidogenic enzymes, thereby reducing testosterone biosynthesis (47). Certain metals, particularly Cd and Hg, may additionally interact with estrogenic or other hormone-responsive signaling pathways, further perturbing endocrine homeostasis (48). Thus, heavy-metal-induced reproductive injury reflects the combined effects of direct cellular and genetic toxicity and, in some cases, endocrine disruption, ultimately compromising the testicular microenvironment, spermatogenesis, and male fertility (49).

## 3 Effects of EDCs exposure during critical developmental windows

Early development represents a particularly vulnerable period for endocrine disruption because reproductive organs, germ cells, steroidogenic pathways, and the HPG axis undergo rapid and highly coordinated differentiation. Rather than simply producing transient hormonal perturbations, exposure to EDCs during these developmental windows may permanently alter the developmental trajectory of the male reproductive system, with phenotypic consequences emerging months or even decades after the initial exposure (104). During fetal life, in particular, androgen-dependent sexual differentiation establishes the anatomical and functional foundation of male reproductive capacity; disruption of this hormonal environment has been linked to cryptorchidism, hypospadias, impaired spermatogenesis and reduced fertility later in life (105).

### 3.1 Prenatal and perinatal exposure

The fetal period is arguably the most critical developmental window for male reproductive toxicity because testicular differentiation, Leydig-cell steroidogenesis, Sertoli-cell development and genital differentiation are highly dependent on precisely timed androgen signaling. EDCs with estrogenic, anti-androgenic or steroidogenic activity may interfere with these processes through multiple mechanisms, including altered androgen receptor signaling, suppression of testosterone biosynthesis, disruption of steroidogenic enzymes, oxidative stress and epigenetic reprogramming. Recent evidence supports the association between maternal EDC exposure and congenital male reproductive abnormalities. A 2024 systematic review and meta-analysis incorporating 46 studies found that maternal EDC exposure was associated with increased risks of hypospadias (adjusted OR 1.26, 95% CI 1.18–1.35) and cryptorchidism (adjusted OR 1.37, 95% CI 1.19–1.57), although associations varied substantially according to the chemical class and outcome assessed (106).

Phthalates provide one of the most extensively investigated examples. Prenatal exposure to several anti-androgenic phthalates, particularly di(2-ethylhexyl) phthalate (DEHP), dibutyl phthalate (DnBP), benzyl butyl phthalate (BzBP) and diethyl phthalate (DEP), has been associated in human studies with shorter anogenital distance (AGD), an established biomarker of androgen-dependent fetal genital development. A recent comprehensive review of male reproductive health similarly concluded that prenatal phthalate exposure is associated with alterations in AGD, reproductive tract development and circulating reproductive hormones (8). Nevertheless, the epidemiological evidence is not uniformly positive. For example, a 2025 prospective study reported no association between prenatal phthalate exposure and AGD in male infants at 12 months, highlighting the influence of exposure timing, chemical mixtures, population characteristics and outcome assessment on observed associations (107). Thus, AGD should be interpreted as an indicator of disrupted androgen action rather than as definitive evidence of subsequent infertility.

The consequences of prenatal exposure may also extend beyond structural abnormalities to the functional programming of the adult testis. Experimental studies indicate that fetal EDC exposure can alter Sertoli-cell proliferation, Leydig-cell steroidogenesis, germ-cell survival and the establishment of the testicular microenvironment, potentially resulting in reduced sperm production and impaired sperm quality in adulthood. Importantly, these effects may persist even after the chemical has been eliminated, supporting the concept of developmental programming rather than ongoing direct toxicity. Epigenetic reprogramming is considered an important mechanistic bridge between transient developmental exposure and persistent reproductive phenotypes. DNA methylation, histone modifications, chromatin remodeling and non-coding RNAs can alter the expression of genes involved in steroidogenesis, germ-cell differentiation, oxidative stress responses and testicular function. Recent reviews further suggest that PFAS exposure during critical developmental periods may modify DNA methylation and other sperm-epigenetic signatures, providing a potential molecular link between early-life exposure and later reproductive dysfunction (108).

Importantly, developmental programming is not restricted to the reproductive tract itself. The developing hypothalamus is also susceptible to endocrine perturbation, and alterations in the neural circuits controlling gonadotropin-releasing hormone (GnRH) secretion may subsequently modify the timing and progression of puberty. Thus, prenatal EDC exposure may affect male reproductive health through a coordinated disruption of the brain–pituitary–testis axis, rather than through direct testicular toxicity alone.

### 3.2 Childhood and puberty

Childhood and puberty represent important developmental windows because maturation of the HPG axis and testicular development occur during this period. EDC exposure may interfere with pubertal development; however, current epidemiological evidence remains inconsistent. A systematic review and meta-analysis of 23 studies found no consistent association between prenatal or postnatal exposure to the examined EDCs and pubertal timing in boys or girls, and no specific exposure window could be definitively identified as particularly susceptible (109). More recent reviews similarly indicate that the effects of BPA, phthalates, PFAS and pesticides on puberty vary according to the chemical, exposure timing, dose and sex, with both earlier and delayed puberty reported in different populations (110, 111). Therefore, although puberty is considered a potentially sensitive window for endocrine disruption, current human evidence is insufficient to conclude that childhood EDC exposure consistently alters pubertal timing or causes subsequent adult male reproductive dysfunction.

EDCs may also affect reproductive health indirectly through metabolic pathways. Some EDCs, termed “obesogens,” can interfere with adipogenesis, lipid metabolism and energy homeostasis and have been associated with obesity-related outcomes, particularly following early-life exposure (112). Because obesity itself is associated with altered reproductive hormone profiles and impaired male reproductive function, metabolic disturbances may represent an additional pathway linking early-life EDC exposure to reproductive health. However, the extent to which this pathway contributes to adult male infertility after childhood exposure remains to be established.

### 3.3 Multigenerational and transgenerational effects

Multigenerational and transgenerational effects should be distinguished when evaluating the long-term consequences of EDC exposure. Following maternal exposure during pregnancy, the F1 fetus and the F2 germline are directly exposed; therefore, effects observed in F1 and F2 are considered multigenerational, whereas persistence of a phenotype in the unexposed F3 generation is required to demonstrate a maternal-line transgenerational effect. Following paternal exposure, F1 is exposed through the paternal germline, making F2 the first generation without direct exposure.

Evidence for EDC-induced transgenerational effects comes predominantly from animal studies. Vinclozolin, an anti-androgenic fungicide, has been reported to induce persistent reproductive abnormalities and impaired spermatogenesis across subsequent generations in rodents following embryonic exposure (113). Alterations in sperm DNA methylation, non-coding RNAs and histone retention have been proposed as potential mechanisms underlying these effects (114). However, findings have not been completely consistent across experimental studies, and differences in species, strain, exposure dose and experimental design may contribute to discrepant results (115). Some evidence also suggests that paternal EDC exposure can induce reproductive, metabolic and developmental effects in offspring, potentially involving sperm-mediated epigenetic alterations (7). Nevertheless, evidence for true transgenerational inheritance in humans remains insufficient, and the relevance of these experimental findings to human male reproductive health requires further investigation.

Taken together, current evidence supports a developmental-window model of EDC-associated male reproductive toxicity, in which fetal sex differentiation, the perinatal period, childhood and puberty represent partially overlapping but biologically distinct periods of susceptibility. The consequences of exposure are determined not simply by chemical concentration but by the interaction among timing, dose, duration, chemical class, mixture composition, sex, genetic background and developmental state. This may explain why low-dose exposure can produce substantial effects during sensitive developmental periods while producing weaker or qualitatively different effects in adults.

## 4 Discussion

Male reproductive health has become a globally significant public health concern. Since the mid-1970s, sperm concentrations in Western countries have declined by more than 50%, with an average annual decline of 1.4% between 1973 and 2011 (116). This trend is difficult to attribute solely to genetic factors or lifestyle changes, and environmental factors are widely considered to be important drivers. Over the past decade, population-based epidemiological studies have consistently revealed associations between EDCs exposure and adverse reproductive outcomes, including impaired semen quality, abnormal hormone levels, and reduced fertility (117).

The detrimental effects of EDCs on the male reproductive system are not mediated through a single pathway but involve multi-level interference ranging from endocrine regulation to organelle function and from gene expression to the epigenome (118). In terms of hormonal regulation, EDCs interfere with the normal function of the HPG axis by mimicking or antagonizing sex hormone actions, which has become one of the most widely recognized core mechanisms. BPA can exert antagonistic effects by binding to the AR, and certain pesticide metabolites (e.g., DDE) also possess anti-androgenic activity. Notably, BPA alternatives such as BPS and BPF retain endocrine-disrupting activity, and some analogues even exhibit toxicity comparable to or greater than that of BPA (119). At the organelle level, mitochondrial dysfunction and OS represent another core pathway through which EDCs induce germ cell injury. Studies have shown that EDCs can trigger excessive production of mitochondrial ROS, leading to energy metabolism disorders, lipid peroxidation, and sperm DNA damage (120). In addition, disruption of the BTB integrity is an important aspect of EDC reproductive toxicity (121). At the epigenetic level, animal experimental evidence indicates that EDCs can induce epigenetic reprogramming via DNA methylation, histone modifications, and non-coding RNA regulation (55, 122). This not only results in diminished sperm quality in exposed individuals but may also give rise to transgenerational or even multigenerational effects. However, validation of such transgenerational effects in humans remains elusive (123).

In real-world settings, humans are not exposed to a single EDC but rather to complex mixtures of multiple chemicals simultaneously. This cocktail effect poses a fundamental challenge to the current regulatory framework based on single-compound assessments (124). Emerging human epidemiological evidence also supports the relevance of mixture effects. In a 2025 population-based cohort of 841 young men, prenatal exposure to a mixture of PFAS, phthalate metabolites and triclosan was associated with lower sperm concentration and total sperm count, although the confidence intervals included the null and the authors noted potential exposure misclassification. The estimated associations appeared to be driven primarily by PFAS exposure (125). Similarly, a targeted exposomics study simultaneously quantified 55 EDCs in urine and identified a mixture of bisphenols, parabens, benzophenones and phthalate metabolites associated with diminished sperm quality, illustrating the value of assessing co-exposure rather than evaluating chemicals in isolation (126). These findings indicate that mixture toxicity should not simply be regarded as the sum of individual chemical effects. Depending on their molecular targets, chemicals within a mixture may act additively, synergistically or antagonistically, while chemicals with different biological half-lives may also represent different components of the same exposure profile. Furthermore, non-monotonic dose-response relationships (NMDRs) challenge the conventional assumption that increasing exposure necessarily produces proportionally greater toxicity. EDCs may produce U-shaped or inverted U-shaped responses because low concentrations can interact with endogenous hormone signaling, receptor sensitivity, negative feedback regulation, receptor desensitization or metabolic compensation in ways that differ from high-dose exposure (127). This issue is particularly relevant to male reproductive toxicology because physiological endocrine systems operate within narrow concentration ranges and are intrinsically regulated by feedback loops. Consequently, the absence of an effect at a high experimental dose does not necessarily exclude an effect at a lower, environmentally relevant concentration. For example, NMDRs have been repeatedly reported for BPA and other endocrine-active chemicals, although the reproducibility, biological plausibility and regulatory implications of individual NMDRs remain subjects of ongoing debate (128, 129).

The embryonic and neonatal periods represent highly sensitive windows for male reproductive system development, and EDC exposure during these stages can exert programmed, lifelong impacts (130). The historical lesson of prenatal diethylstilbestrol (DES) exposure leading to cryptorchidism and hypospadias in male offspring has long served as a warning (131). Subsequent studies have found that prenatal phthalate exposure can shorten AGD in male infants, reflecting suppressed androgen signaling and impaired testicular function (132). These findings underscore the urgency of preventing EDC exposure during early life, and preventing exposure during critical developmental windows may have far-reaching significance.

The exposome shifts the research focus from individual chemicals toward the totality of environmental exposures experienced throughout the life course. In male reproductive research, exposome-based approaches can integrate chemical exposures with diet, smoking, occupation, air pollution, psychosocial factors and other environmental determinants, thereby more closely reflecting real-world exposure conditions. Importantly, chemical exposomics is also beginning to move beyond predefined target lists. For instance, a recent human preconception study detected multiple classes of xenobiotics—including PFAS, parabens, UV filters, plastic additives and antimicrobials—in paired biological fluids, including reproductive fluids, suggesting that relying solely on serum or urine for exposure assessment may underestimate the chemical complexity relevant to reproductive tissues (133). This is particularly critical for male reproductive toxicology, because circulating or urinary concentrations may not accurately represent the local exposure milieu at the testis, epididymis, or seminal microenvironment. Therefore, future studies should increasingly combine repeated biomonitoring with targeted and non-targeted high-resolution mass spectrometry to characterize both known and unrecognized reproductive toxicants. Furthermore, the rapid development of multi-omics technologies offers an additional opportunity to link exposure profiles to biological effects. Integrating transcriptomics, epigenomics, proteomics, metabolomics, and single-cell sequencing can help identify exposure-responsive pathways, cell-type-specific alterations, and potential molecular mediators between environmental exposure and impaired spermatogenesis.

Although a substantial body of evidence supports an association between EDC exposure and male infertility, important gaps remain in this field. Most existing human studies employ cross-sectional designs, making it difficult to establish causality. Meanwhile, EDC exposure assessment predominantly relies on single measurements in blood or urine, which cannot accurately reflect the actual exposure levels within target organs such as the testis. Moreover, EDCs typically exist as mixed exposures, and their interactions and synergistic effects with lifestyle factors such as diet, smoking, and psychological stress remain to be thoroughly elucidated. Future research should further optimise exposure assessment methodologies, explore exposure monitoring strategies at the target organ level, establish risk assessment frameworks applicable to EDC mixtures, and integrate novel technologies such as organoids and multi-omics to gain deeper insights into the molecular mechanisms by which EDCs impair spermatogenesis and reproductive endocrine function.
